# Marine alkaloids as the chemical marker for the prey–predator relationship of the sponge *Xestospongia* sp. and the nudibranch *Jorunna funebris*

**DOI:** 10.1007/s42995-021-00096-w

**Published:** 2021-03-29

**Authors:** Qihao Wu, Song-Wei Li, Nicole J. de Voogd, Hong Wang, Li-Gong Yao, Yue-Wei Guo, Xu-Wen Li

**Affiliations:** 1 State Key Laboratory of Drug Research, Shanghai Institute of Materia Medica, Chinese Academy of Sciences, Shanghai, 201203 China; 2 Open Studio for Druggability Research of Marine Natural Products, Pilot National Laboratory for Marine Science and Technology (Qingdao), Qingdao, 266237 China; 3 College of Pharmaceutical Science and Collaborative Innovation Center of Yangtze River Delta Region Green Pharmaceuticals, Zhejiang University of Technology, Hangzhou, 310014 China; 4 Nanjing University of Chinese Medicine, Nanjing, 210023 China; 5 National Museum of Natural History, PO Box 9517, 2300 RA Leiden, Netherlands; 6 Institute of Environmental Sciences, Leiden University, PO Box 9518, 2300 RA Leiden, Netherlands

**Keywords:** Marine alkaloids, *Jorunna funebris*, *Xestospongia* sp. Chemical marker

## Abstract

The dietary relationship study between marine sponge *Xestospongia* sp. and its nudibranch predators *Jorunna funebris* based on the discovery of isoquinolinequinones has long been studied. In this study, chemical investigation of the sponge *Xestospongia* sp. and nudibranch *J. funebris* from the South China Sea yielded a new marine alkaloid neopetroside C (**1**), together with nine known alkaloids (**2**–**10**). The chemical structures of all the compounds were elucidated by extensive spectroscopic analysis. Neopetroside C (**1**) featured a riboside of nicotinic acid with a rare *α*-*N* glycosildic linkage and an acyl residue of (*Z*)-2-methylbut-2-enoic acid attached to C-5′. The plausible chemical ecology relationship between sponge *Xestospongia* sp. and its nudibranch predator *J. funebris* was proposed based on the biogenetic relationship of the common marine alkaloids. The observation of two structural fragments, (*Z*)-2-methylbut-2-enoyloxy and trigonelline groups in both sponge and nudibranch, indicated that nudibranch might uptake chemicals from sponge and then modify and transform them into chemical weapons to defend against predators.

**Supplementary Information:**

The online version contains supplementary material available at 10.1007/s42995-021-00096-w.

## Introduction

In the past several decades, different species of marine sponges have been investigated for their bioactive secondary metabolites. A number of studies have indicated that marine sponges are one of the richest sources of marine natural products with potential therapeutic application (Liu et al. 2019; Wu et al. 2019a, b; Zhu et al. 2019). The marine sponges of the genus *Xestospongia* are widely distributed in the South China Sea. They are well known as one of the richest sources of diverse bioactive natural products, including brominated polyacetylenes (Yang et al. 2019) and alkaloids (Huang et al. 2016). Some of these metabolites have attracted the attention for their total syntheses and/or biological studies towards drug leads (Liang et al. 2014; Sun et al. 2017).

The marine nudibranchs are known to assimilate small molecules based on prey-predator relationship from other low-grade organisms such as sponges for chemical defense against harsh marine living environments (Bornancin et al. 2017; Dean and Prinsep 2017; Puglisi et al. 2019; Wu et al. 2020). The marine nudibranch *Jorunna funebris* (Mollusca: Gastropoda: Opisthobranchia: Nudibranchia: Kentrodorididae) has been proven to feed on sponge of the genus *Xestospongia*, by the discovery of isoquinolinequinones as the chemical marker from both nudibranch *J. funebris* and its sponge-prey *Xestospongia* sp. (Huang et al. 2016). As part of our ongoing project on searching for novel bioactive compounds from Hainan marine sponges (Han et al. 2018; Huang et al. 2016; Wu et al. 2019a, b; Xue et al. 2017; Yang et al. 2019) and the chemical ecology study between sponges and their nudibranch predator, we made a collection of the sponge *Xestospongia* sp. and its possible nudibranch predator *J. funebris* from the same location (Xidao Island, Hainan Province). In this paper, we described the discovery of 10 marine alkaloids, as well as the dietary relationship between the sponge *Xestospongia* sp. and its predator *J. funebris* based on those marine alkaloids.

## Results

**Fig. 1:**
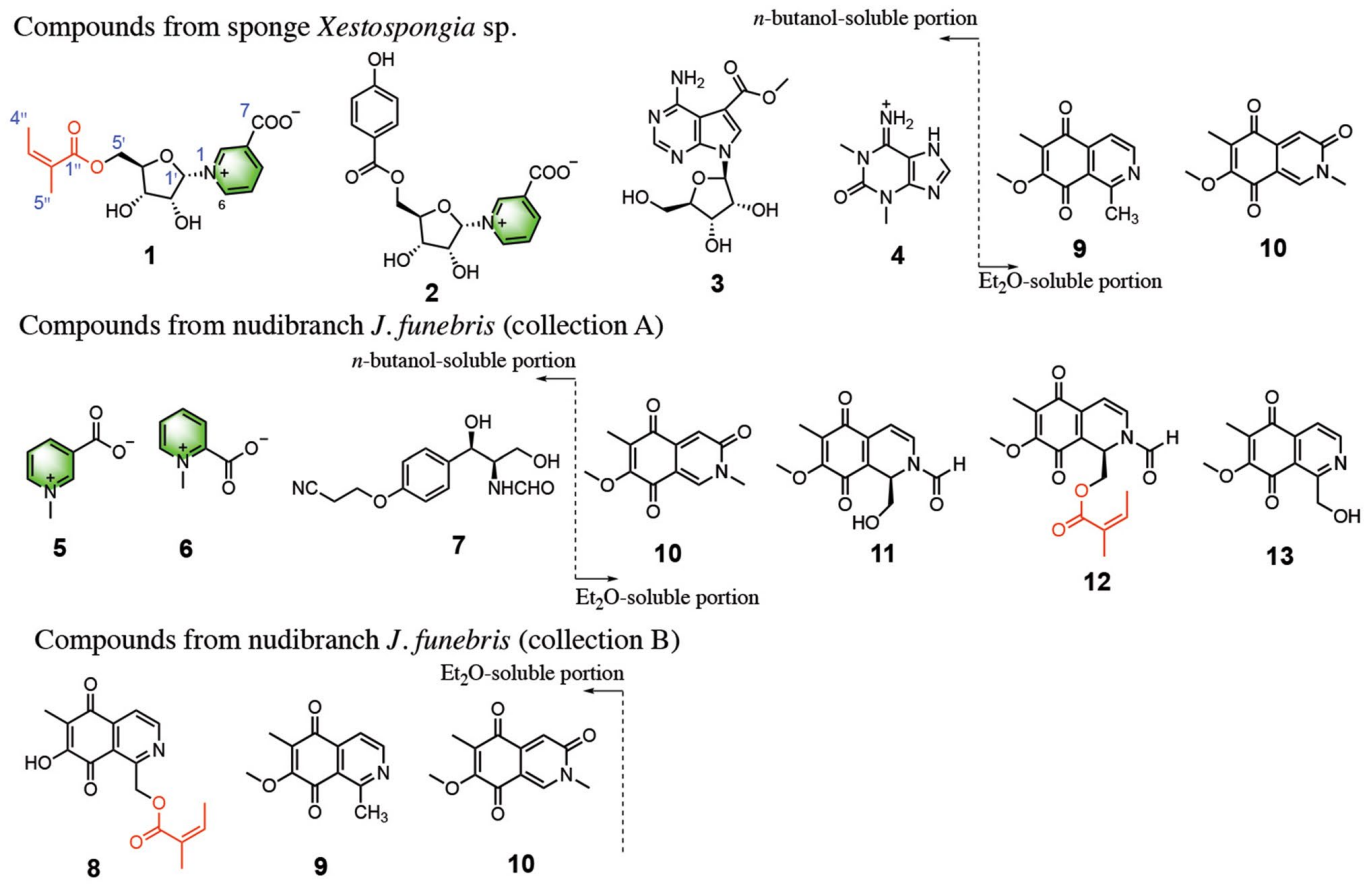
Chemical structures of compounds **1**–**13**. Compounds **1**–**10** were reported in this paper, compounds **11**–**13** were reported previously (Huang et al. 2016). The moieties are marked in the same color among all the compounds

Chemical investigation of the sponge *Xestospongia* sp. and two nudibranchs *J. funebris* led to the isolation of a previously unreported pyridine nucleoside (**1**) along with nine known alkaloids (**2**–**10**) (Fig. 1). Herein, we describe the isolation, structure elucidation, and their possible biosynthetic origin influenced by a prey–predator relationship.

The frozen title sponge (*Xestospongia* sp.) was cut into pieces and exhaustively extracted by acetone. The *n*-butanol-soluble portion of the acetone extract was chromatographed repeatedly over MCI and RP-HPLC to yield **1**–**4** (Fig. 1). The Et_2_O-soluble portion of the acetone extract was repeatedly purified on column chromatography (CC) of silica gel, Sephadex LH-20, reversed-phase-C18 (RP-C18) and RP-HPLC to afford **9** and **10** (Fig. 1). Among them, the known compounds were readily identified as neopetroside A (**2**) (Shubina et al. 2015), 5-(methoxycarbonyl)tubercidin (**3**) (Wang et al, 2016), 1,3-dimethylisoguaninium (**4**) (Jeong et al. 2003), 7-methoxy-1,6-dimethyl-5,8-dihydroisoquinoline-5,8-dione (**9**) (Suwanborirux et al. 2003), and mimosamycin (**10**) (Kesteleyn and Kimpe 2000) by comparison of their NMR spectroscopic data and optical rotation with those reported in the literature.

The frozen animals *J. funebris* were cut into pieces and exhaustively extracted by acetone. For collection A (No. 14X-219 collected in 2014), the *n*-butanol-soluble portion of the acetone extract was repeatedly chromatographed to yield pure compounds **5**–**7**. For collection B (No. 18XD-200, collected in 2018), the Et_2_O-soluble portion of the acetone extract gave **8**–**10**. The known compounds were identified as trigonelline (**5**) (Poulin et al. 2018), homarine (**6**) (Poulin et al. 2018), bursatellin (**7**) (Cimino et al. 1987), *O*-demethylrenierone (**8**) (Plubrukarn et al. 2003), 7-methoxy-1,6-dimethyl-5,8-dihydroisoquinoline-5,8-dione (**9**) (Suwanborirux et al. 2003), and mimosamycin (**10**) (Kesteleyn and Kimpe 2000).

Neopetroside C (**1**) was obtained as a brown amorphous solid, [*α*] + 66 (*c* 0.1, MeOH). Its molecular formula, C_16_H_19_NO_7_, was established by HRESIMS (*m/z* 338.1240, [M + H]^+^, calcd for C_16_H_20_NO_7_, 338.1234), indicating eight degrees of unsaturation. The ^13^C NMR spectrum displayed 16 carbon signals which, in combination with DEPT and HSQC spectra, can be categorized as two methyls, one sp^3^ methylene, four sp^3^ methines, five sp^2^ methines, four sp^3^ quaternary carbons. Detailed analysis of spectroscopic data of compound **1** revealed the presence of a ribose moiety (*δ*_H_ 4.36, *δ*_H_ 4.48, *δ*_C_ 64.3, CH_2_; *δ*_H_ 6.47, *δ*_C_ 98.0, CH; *δ*_H_ 4.76, *δ*_C_ 73.6, CH; *δ*_H_ 4.28, *δ*_C_ 72.6, CH; *δ*_H_ 4.90, *δ*_C_ 87.3, CH) (Shubina et al. 2015), the signals of the carbons and protons of the aromatic ring (*δ*_H_ 9.27, *δ*_C_ 143.7, CH; *δ*_C_ 138.6, qC; *δ*_H_ 8.95, *δ*_C_ 147.3, CH; *δ*_H_ 8.07, *δ*_C_ 127.2, CH; *δ*_H_ 8.94, *δ*_C_ 143.1, CH), and two downfield quaternary carbons at *δ*_C_ 167.1 and 168.7 (Table 1).

**Table 1:** ^1^H (500 MHz) and ^13^C NMR (125 MHz) data (*δ* in ppm, type) of **1**^a^ and **2**^b^ recorded in CD_3_OD

No.	**1**		**2**	
	*δ*_H_ mult (*J* in Hz)	*δ*_C_	*δ*_H_ mult (*J* in Hz)	*δ*_C_
2	9.27 s	143.7 CH	9.28 s	144.3 CH
3	–	138.6 qC	–	139.5 CH
4	8.94 d (7.0)	147.3 CH	8.94 dd (8.0, 1.4)	147.9 CH
5	8.07 t (7.0)	127.2 CH	8.06 dd (8.0, 6.2)	127.8 CH
6	8.95 dd (7.0)	143.1 CH	8.96 dd (6.2, 1.4)	143.7 CH
7	–	167.1 qC	–	167.2 qC
1′	6.47 d (5.5)	98.0 CH	6.50 d (5.3)	98.6 CH
2′	4.76 t (4.5)	73.6 CH	4.79 m	74.4 CH
3′	4.28 dd (4.5,3.5)	72.6 CH	4.33 dd (3.8, 4.5)	73.3 CH
4′	4.90 dt (4.5, 3.5)	87.3 CH	4.96 ddd (3.8, 3.6, 4.6)	88.0 CH
5′a	4.36 dd (12.0, 4.5)	64.3 CH_2_	4.50 dd (4.6, 12.2)	65.3 CH_2_
5′b	4.48 dd (12.0, 3.5)		4.59 dd (3.6, 12.2)	
1′′	–	168.7 qC	–	122.3 qC
2′′	–	128.6 qC	7.93, d (8.8)	133.6 CH
3′′	6.19 qq (7.5, 1.5)	140.2 CH	6.68, d (8.8)	117.0 CH
4′′	2.01 dq (7.5, 1.5)	16.1 CH_3_	–	164.5 qC
5′′	1.95 quint (1.5)	20.7 CH_3_	6.68, d (8.8)	117.0 CH
6′′	–	–	7.93, d (8.8)	133.6 CH
7′′	–	–	–	168.2 qC

^a^Assignments were deduced by analysis of 1D and 2D NMR spectra

^b^Chemical shifts of **2** were reported in the reference (Shubina et al. 2015)

**Fig. 2:**
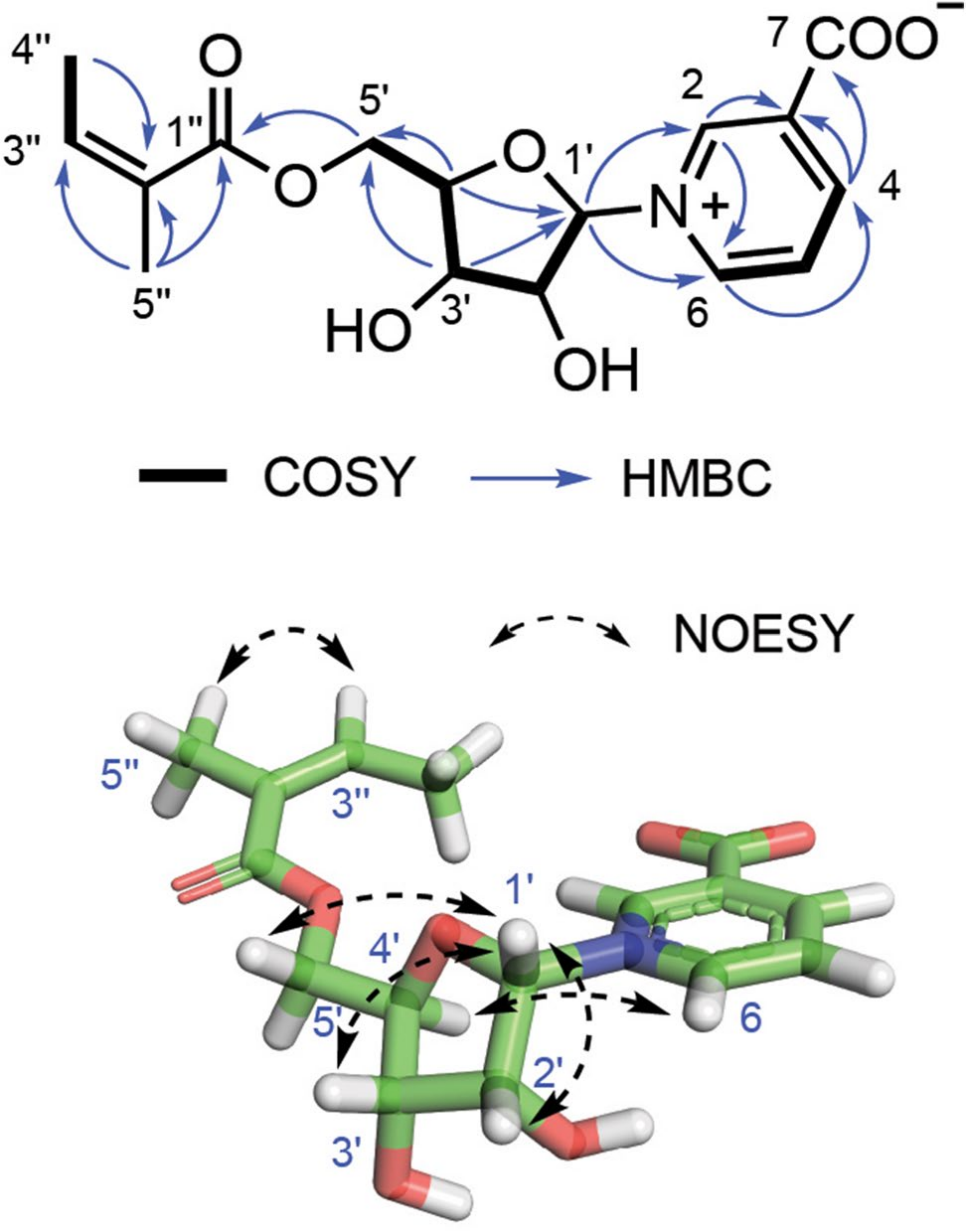
^1^H-^1^H COSY, key HMBC, and NOESY correlations of compound 1

**Fig. 3:**
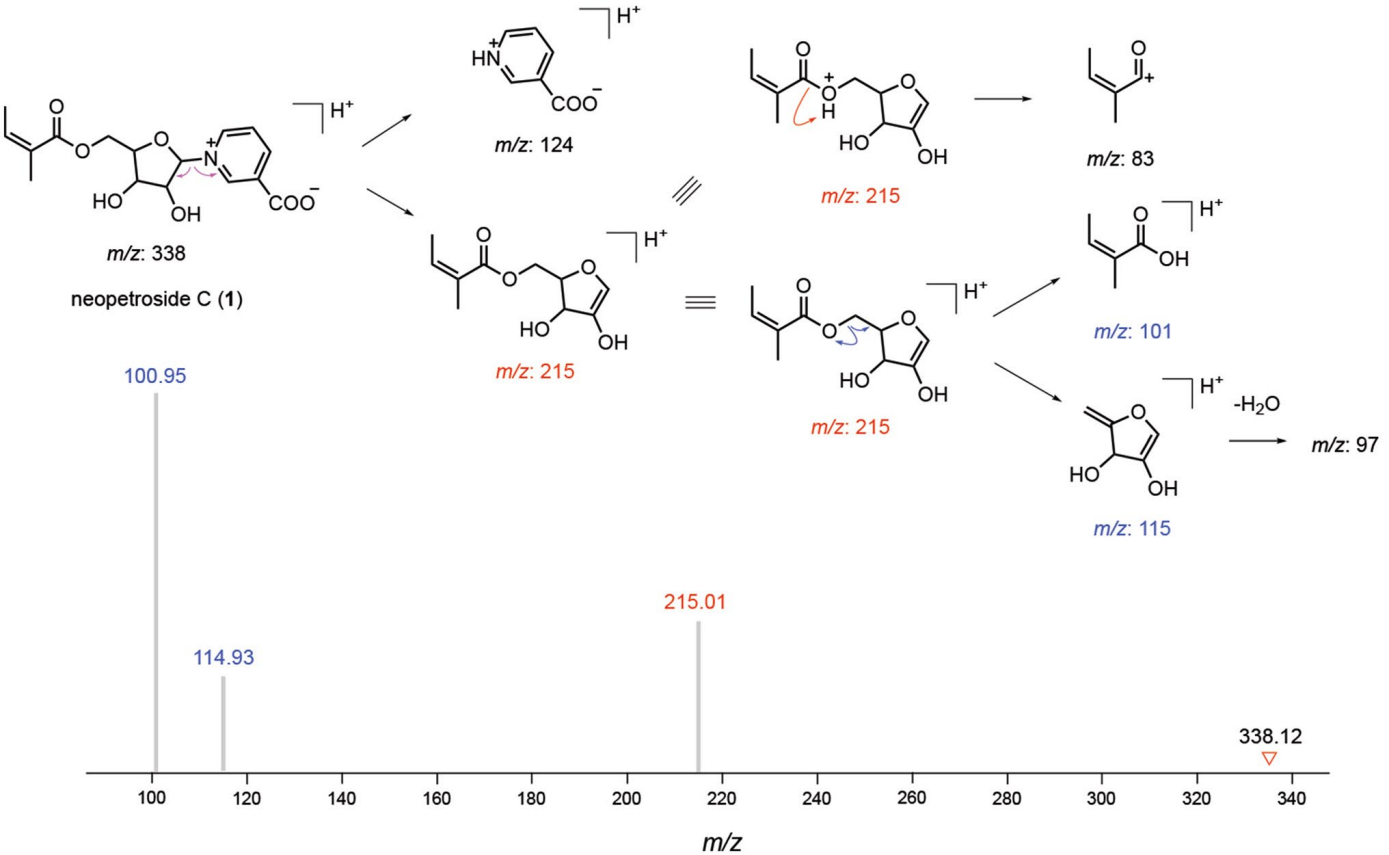
Observed product ions during MS^*n*^ fragmentation experiments of compound **1** and assignment of the molecular ion peaks

The above structural features were reminiscent of co-occurring neopetroside A (**2**), which was firstly isolated from the sponge of the genus *Neopetrosia* (Shubina et al. 2015). A careful comparison of the NMR data of these two compounds revealed that they differed only by the substitution at C-5′ (Fig. 1). The remaining signals of one trisubstituted double bond (*δ*_H_ 6.19, *δ*_C_ 140.2, CH; *δ*_C_ 128.6, qC) and two methyls (*δ*_H_ 1.95, *δ*_C_ 20.7, CH_3_; *δ*_H_ 2.01, *δ*_C_ 16.1, CH_3_) were then assigned as a (*Z*)-2-methylbut-2-enoate moiety, which was determined by the presence of a HMBC cross-peak between Me-5" and H-3". It was further supported by comparing the spectroscopic data with those reported in the literature for (*Z*)-2-methylbut-2-enoyloxy-containing natural products (Plubrukarn et al. 2003). The HMBC correlations of the methylene protons H_2_-5′ to the carbonyl C-1′′ indicated that the (*Z*)-2-methylbut-2-enoyloxy group was attached to C-5′ of the sugar moiety in **1**. On the basis of the above findings, the planar structure of **1** was fully established (Fig. 2). The structure of **1** was further confirmed by ESI-MS^*n*^ analysis, showing fragmentations with the loss of functional groups. The yielded ESI-MS^*n*^ ion peaks of neopetroside C (**1**) were analyzed and fully assigned as shown in Fig. 3.

The configuration of the ribofuranoside ring in **1** was also determined to be the same as co-occurring **2** by careful interpretation of its NOESY spectrum, with the clear NOE correlations of H-1′/H-3′, H_2_-5′ and H-4′/H-2, H-6 (Fig. 2). Since the absolute configuration of **2** has been previously determined by total synthesis, from a biogenetic point of view, bearing in mind the same signs of the [*α*]_D_ values of **1** (+ 66) and **2** (+ 20) (Shubina et al. 2015), the absolute configuration of compound **1** was assigned as 1′*S*, 2′*R*, 3′*S*, 4′*R*.

## Discussion

**Fig. 4:**
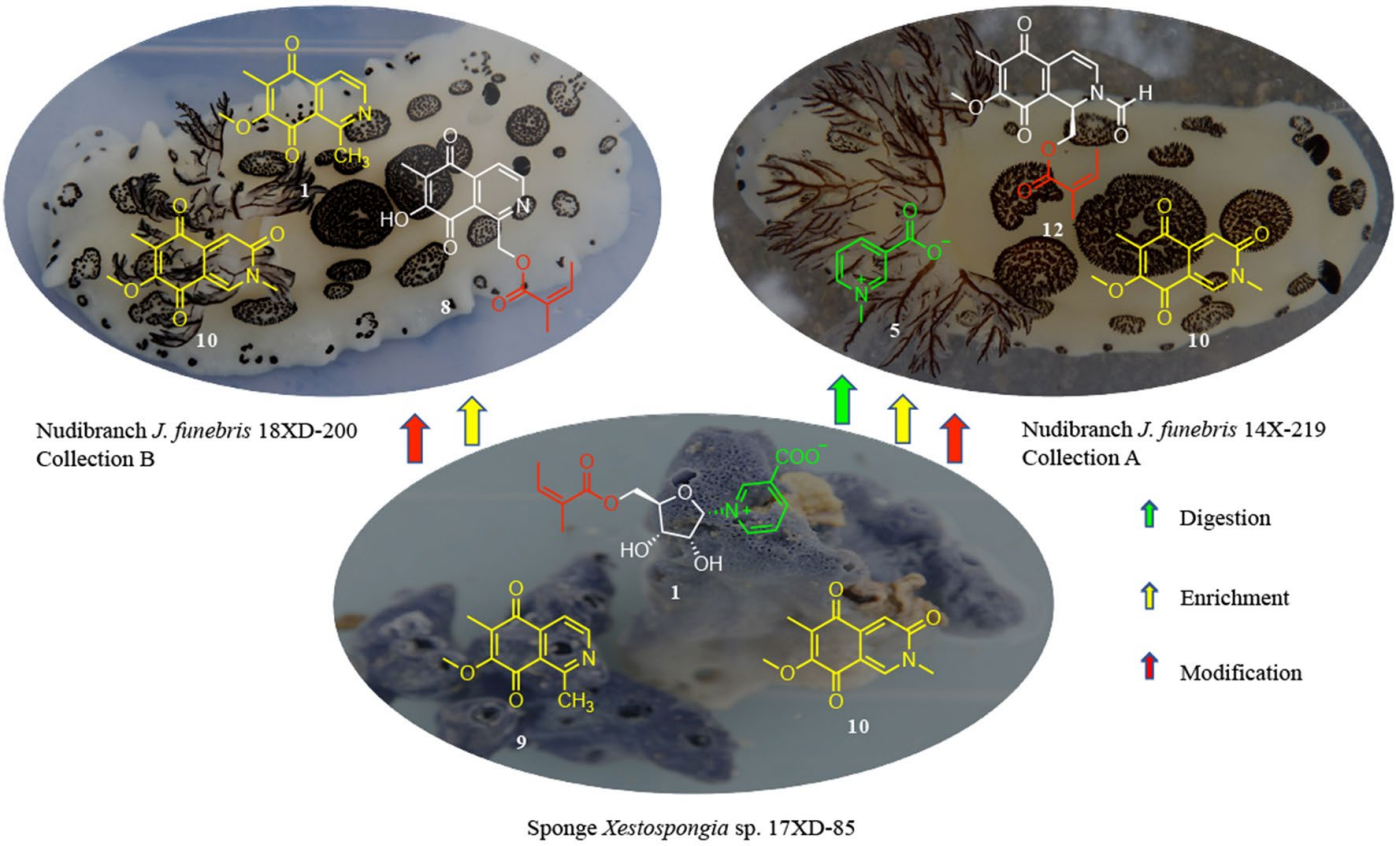
Dietary relationship between nudibranch *J. funebris* and sponge *Xestospongia* sp. uncovered by neopetrosides and isoquinolines as characteristic chemical markers

In this study, a chemical investigation of the two title animals resulted in the establishment of a dietary relationship between nudibranch *J. funebris* and its sponge-prey *Xestospongia* sp. As shown in Fig. 4, by careful comparison of the alkaloids in two nudibranch individuals (collections A and B from the same location in 2014 and 2018, respectively) with those in the sponge *Xestospongia* sp., several common alkaloids or related structural fragments were observed in *J. funebris* nudibranch and *Xestospongia* sp. sponge. To be more specific, common isoquinoline alkaloids (**9** and **10**) were identified from the Et_2_O portions of both *J. funebris* (**10** for collection A; **9** and **10** for collection B) and *Xestospongia* sp., whereas only one alkaloid (**8**) was found in *J. funebris* (collection A). Two rare naturally occurring ribosides (**1** and **2**) as well as their structural fragments (**5**) were discovered from the *n*-BuOH portions of *Xestospongia* sp. and *J. funebris* (collection B), respectively. In addition, the (*Z*)-2-methylbut-2-enoyloxy group was observed in **1**, **8** and **12**. On the basis of these observations, we thus hold the belief that the nudibranch *J. funebris* could accumulate and/or biotransform the sponge-derived metabolites, especially those toxic isoquinolinequinone alkaloids, as its own chemical defensive agents for surviving in the harsh marine living environment. Moreover, it is obvious that the nudibranch *J. funebris* can feed on more than one sponge, such as the aforementioned *Xestospongia* sp., to acquire structurally diverse alkaloid metabolites to apply them as the chemical weapons on different occasions. In turn, such structurally characteristic alkaloids could act as chemical marker to uncover the prey-predator relationship of marine mollusks and sponges.

More intriguingly, to explain the dietary expansion of *J. funebris* in Xidao Island water, an extensive literature search revealed that they may selectively acquire some chemicals from sponges *Xestospongia* sp. to protect themselves from their own predators, the mud crabs. As reported by Dr. Kubanek and her colleagues (Poulin et al. 2018), trigonelline (**5**) and homarine (**6**) in blue crabs' urine induce fear in mud crabs. Thus, we speculated that the *J. funebris* has evolved to use those chemicals to scare mud crabs away. The accumulation of **5** and **6** in *J. funebris* is possibly from a direct dietary relationship since trigonelline and homarine occur in many plants, such as algae (Gebser and Pohnert 2013). However, no such direct evidence has shown that *J. funebris* feed on algae. Back to the compounds, we isolated from the sponge *Xestospongia* sp., the unexpected metabolites **1** and **2** might be the real sources of trigonelline and homarine. The nudibranch selectively ingests *Xestospongia* sp., generating **5** and **6**, by digesting neopetrosides (**1** and **2**).

## Conclusions

In summary, the chemical investigation on one sponge *Xestospongia* sp. and the nudibranch *J. funebris* led to the isolation and identification of ten alkaloids with high chemical diversity. The discovery of one previously unreported compound, namely, neopetroside C (**1**), featured an extremely rare *α*-*N* glycosidic linkage with a nicotinic acid moiety, has added to an extremely diverse and complex array of marine alkaloids which are rapidly expanding. The structures of these compounds, including relative stereochemistry, were elucidated by a combination of detailed spectroscopic analyses and by the comparison of spectroscopic data with those reported in the literature. The absolute configuration of the new metabolite (**1**) was tentatively assigned based on the biogenetic consideration and comparison of its NMR data and optical rotation value with those of the related model compound (**2**). In the future, more chemical ecological research based on predator–prey relationship is crucial to further prove our hypothesis as to the genuine biogenetic relationship of these alkaloids. Further extensive evaluation of the biological activity of these compounds would also be crucial in identifying their pharmacological application.

## Materials and methods

### General experimental procedures

Optical rotations of all the isolates were measured in methanol on a Perkin-Elmer 241MC polarimeter. IR spectrum of **1** was recorded on a Nicolet 6700 spectrometer with KBr pellets. NMR spectra (1D and 2D NMR) were measured on a Bruker DRX-400 and Bruker DRX-500 spectrometer, using the residual methanol signal at *δ*_H_ 3.31 ppm and *δ*_C_ 49.00 ppm as an internal standard for ^1^H NMR and ^13^C NMR, respectively. HR-ESI-MS spectrum of **1** was recorded on Agilent G6250 Q-TOF. Reversed-phase HPLC purification was carried out on an Agilent 1260 series liquid chromatography with a semi-preparative ODS-HG-5 column (5 μm, 250 × 9.4 mm). Silica gel and precoated silica gel plates were used for column chromatography and analytical TLC, respectively. All the chemicals used in this research were obtained from commercial sources. All solvents used for column chromatography were of analytical grade, and solvents used for HPLC were of HPLC grade.

### Biological material, extraction and isolation

#### Biological material

The molluscs and sponges were collected by scuba diving at Xidao Island, Hainan Province, China, at a depth of 15–20 m, and identified by Professor Nicole J. de Voogd. For sponges, samples were collected in May 2017, while molluscs (collections A and B) were collected in May 2014 and May 2018, respectively. The voucher samples are deposited at the Shanghai Institute of Materia Medica, CAS.

#### Extraction and isolation of **1–10**

The lyophilized bodies of sponges *Xestospongia* sp. (dry weight 250 g) were cut into pieces and exhaustively extracted by acetone (20.0 g). The *n*-butanol-soluble portion of the acetone extract (6.1 g) was chromatographed repeatedly over MCI and RP-HPLC to yield **1**–**4**. The Et_2_O-soluble portion of the acetone extract (8.0 g) was repeatedly purified on repeated column chromatography (CC) of silica gel, Sephadex LH-20, RP-C18 CC and RP-HPLC to afford compounds **9** and **10**.

The frozen animals *J. funebris* (dry weight 3.5 g for collection A and dry weight 3.7 g for collection B) were cut into pieces and exhaustively extracted by acetone (0.6 g acetone extract for collection A and 0.7 g acetone extract for collection B). For collection A (No. 14X-219 collected in May 2014), the *n*-butanol-soluble portion of the acetone extract (0.3 g) was repeatedly chromatographed by RP-C18 CC and RP-HPLC to yield **5**–**7**. The chemical investigation of Et_2_O-soluble portion has been reported before (Huang et al. 2016). For collection B (No. 18XD-200, collected in 2018), the Et_2_O-soluble portion of the acetone extract gave **8**–**10**. Chemical investigation of its *n*-butanol-soluble portion failed to isolate any pure compounds.

*Neopetroside C* (**1**), brown amorphous solid, [*α*] + 66 (*c* 0.1, MeOH); for ^1^H and ^13^C NMR spectroscopic data (Table 1); HR-ESI–MS: *m/z* calcd for C_16_H_20_NO_7_ [M + H]^+^: 338.1234; found: 338.1240. See Supplementary Figures S1–S11 in the supplementary document for all the spectra of compound **1**.

## Supplementary Information

Below is the link to the electronic supplementary material.

Supplementary file1 (DOCX 3535 KB)
