# Supplementary material for: Marine alkaloids as the chemical marker for the prey–predator relationship of the sponge Xestospongia sp. and the nudibranch Jorunna funebris
Source: Mar Life Sci Technol. 2021 Mar 29;3(3):375–81. doi: 10.1007/s42995-021-00096-w (PMC10077215; doi:10.1007/s42995-021-00096-w)

**Marine alkaloids as the chemical marker for the prey-predator relationship of the sponge *Xestospongia* sp. and the nudibranch *jorunna funebris***

Qihao Wu,^1,3^ Song-Wei Li,^1,4^ Nicole J. de Voogd,^5,6^ Hong Wang,^3^ Li-Gong Yao,^1^ Yue-Wei Guo*^,1,2,^ and Xu-Wen Li*^,1,2^

^1^State Key Laboratory of Drug Research, Shanghai Institute of Materia Medica, Chinese Academy of Sciences, Shanghai 201203, China

^2^Open Studio for Druggability Research of Marine Natural Products, Pilot National Laboratory for Marine Science and Technology (Qingdao), Qingdao 266237, China

^3^College of Pharmaceutical Science and Collaborative Innovation Center of Yangtze River Delta Region Green Pharmaceuticals, Zhejiang University of Technology, Hangzhou 310014, China

^4^Nanjing University of Chinese Medicine, Nanjing 210023, China

^5^National Museum of Natural History, PO Box 9517, 2300 RA Leiden, Netherlands

^6^Leiden University, Institute of Environmental Sciences, PO Box 9518, 2300 RA Leiden, Netherlands

Corresponding Author

Yue-Wei Guo, ywguo@simm.ac.cn

Xu-Wen Li, xwli@simm.ac.cn

**Supplementary Fig.S1**  ^1^H NMR spectrum (500 MHz) of compound **1** in MeOD.


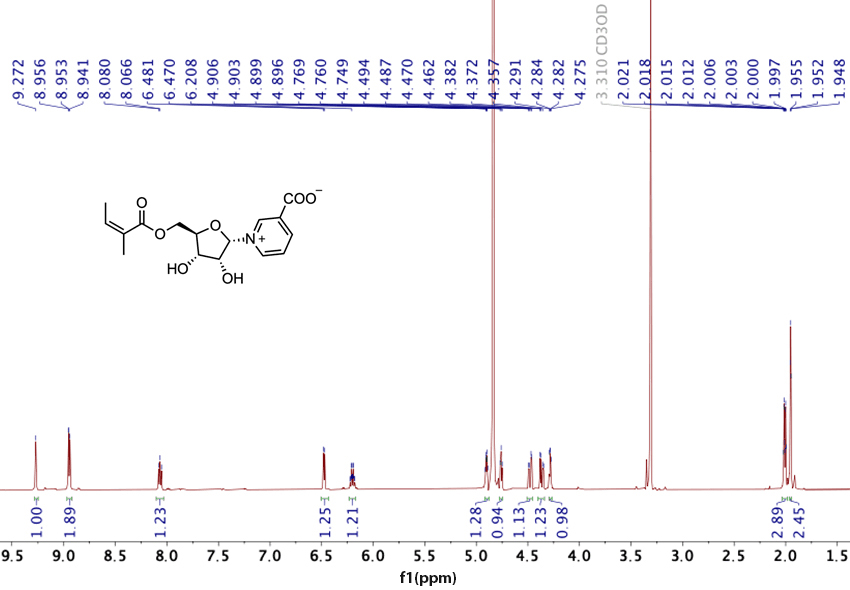


**Supplementary Fig. S2**  ^13^C NMR spectrum (125 MHz) of compound **1** in MeOD.


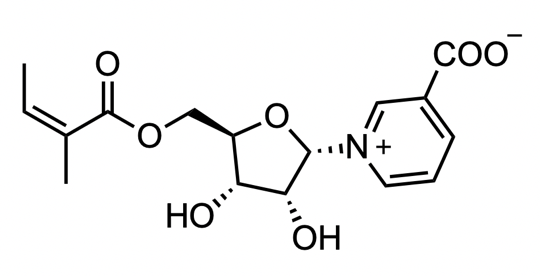

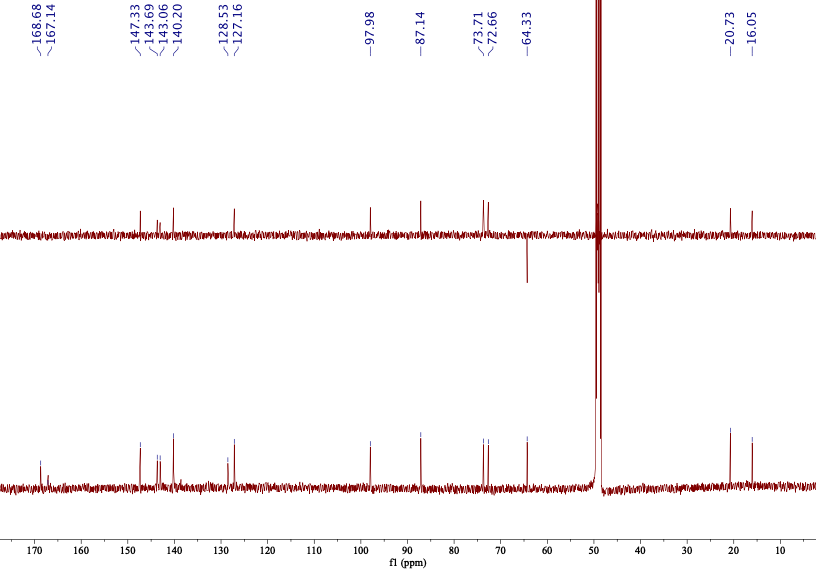


**Supplementary Fig.S3** 1H-1H COSY spectrum (500 MHz) of compound **1** in MeOD.


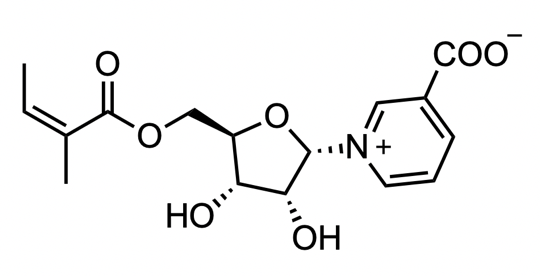

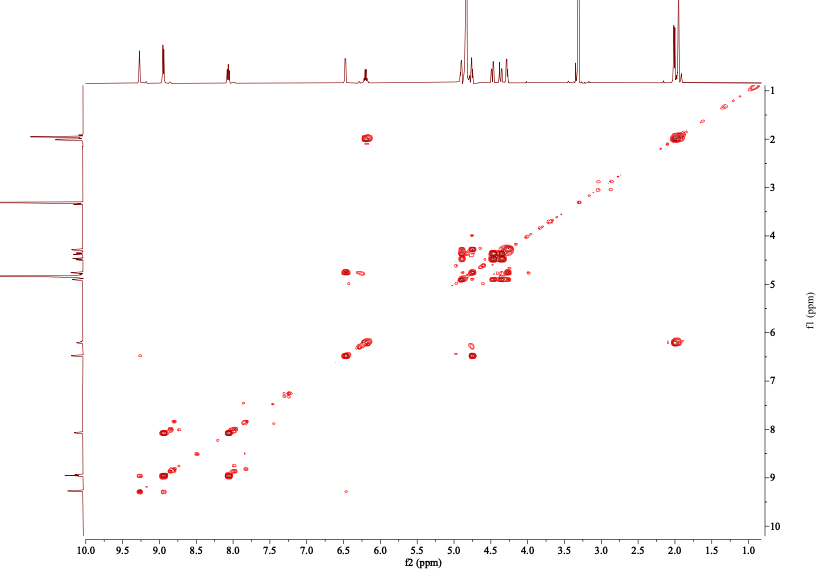


**Supplementary Fig. S4**  HSQC spectrum (500 MHz) of compound **1** in MeOD.


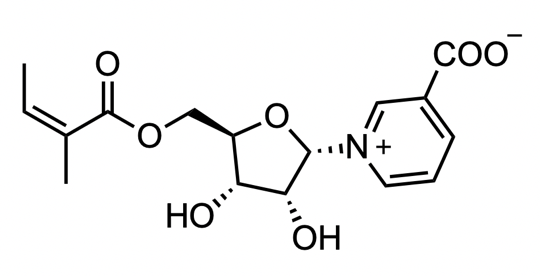

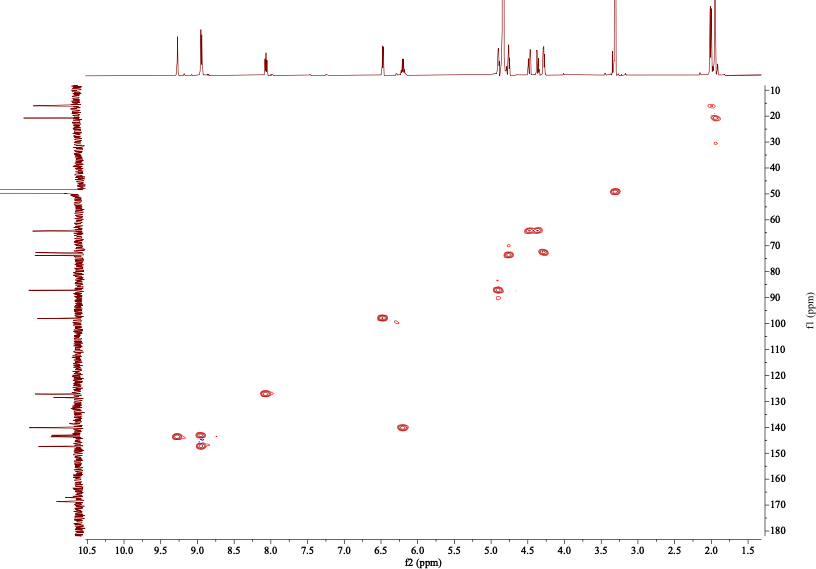


**Supplementary Fig. S5**  HMBC spectrum (500 MHz) of compound **1** in MeOD.


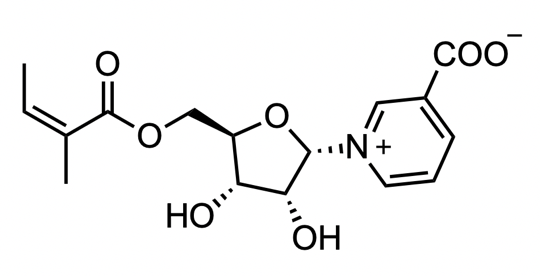

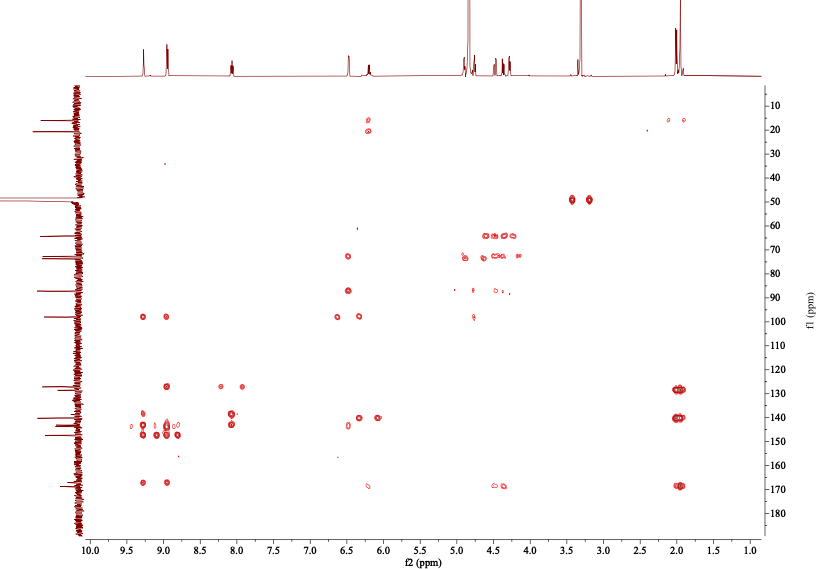


**Supplementary Fig.S6**  NOESY spectrum (500 MHz) of compound **1** in MeOD.


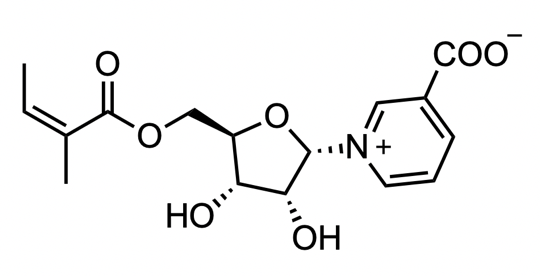

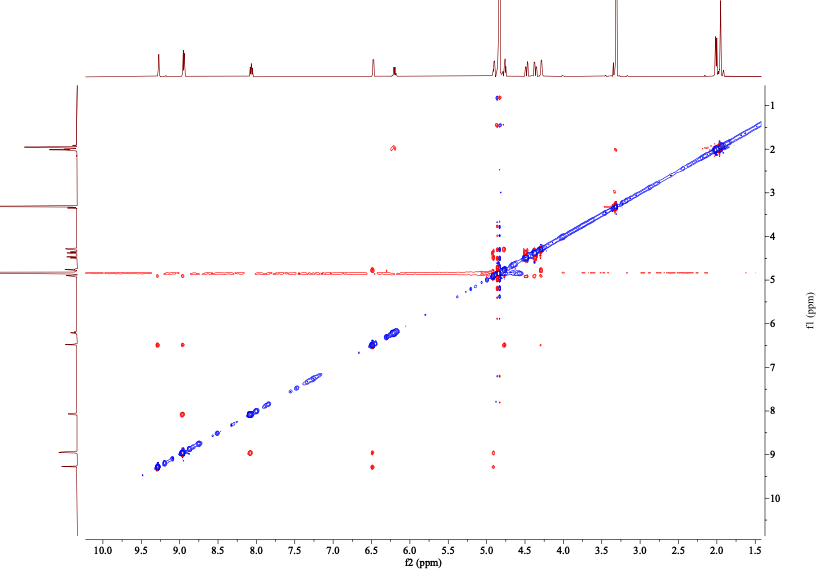


**Supplementary Fig. S7**  IR spectrum of compound **1**.


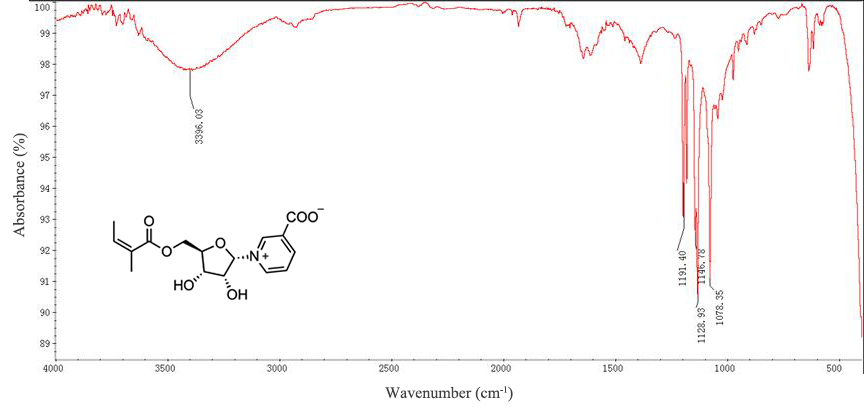


**Supplementary Fig. S8**  HR-ESI-MS spectrum of **1**.


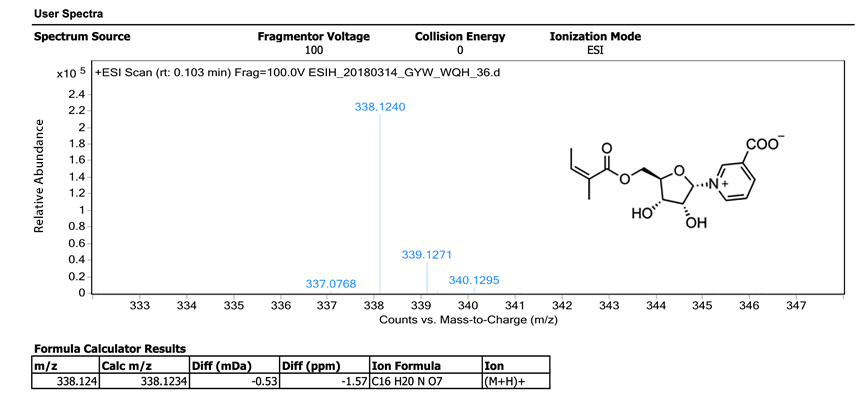


**Supplementary Fig. S9**  ESI-MS/MS spectrum of **1**.


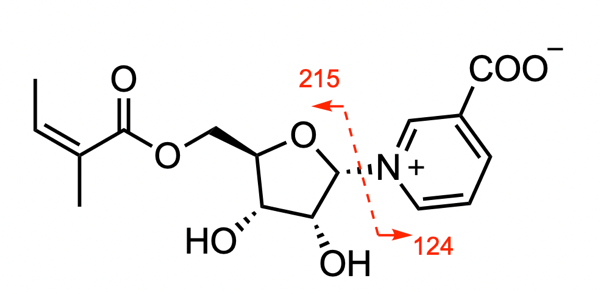

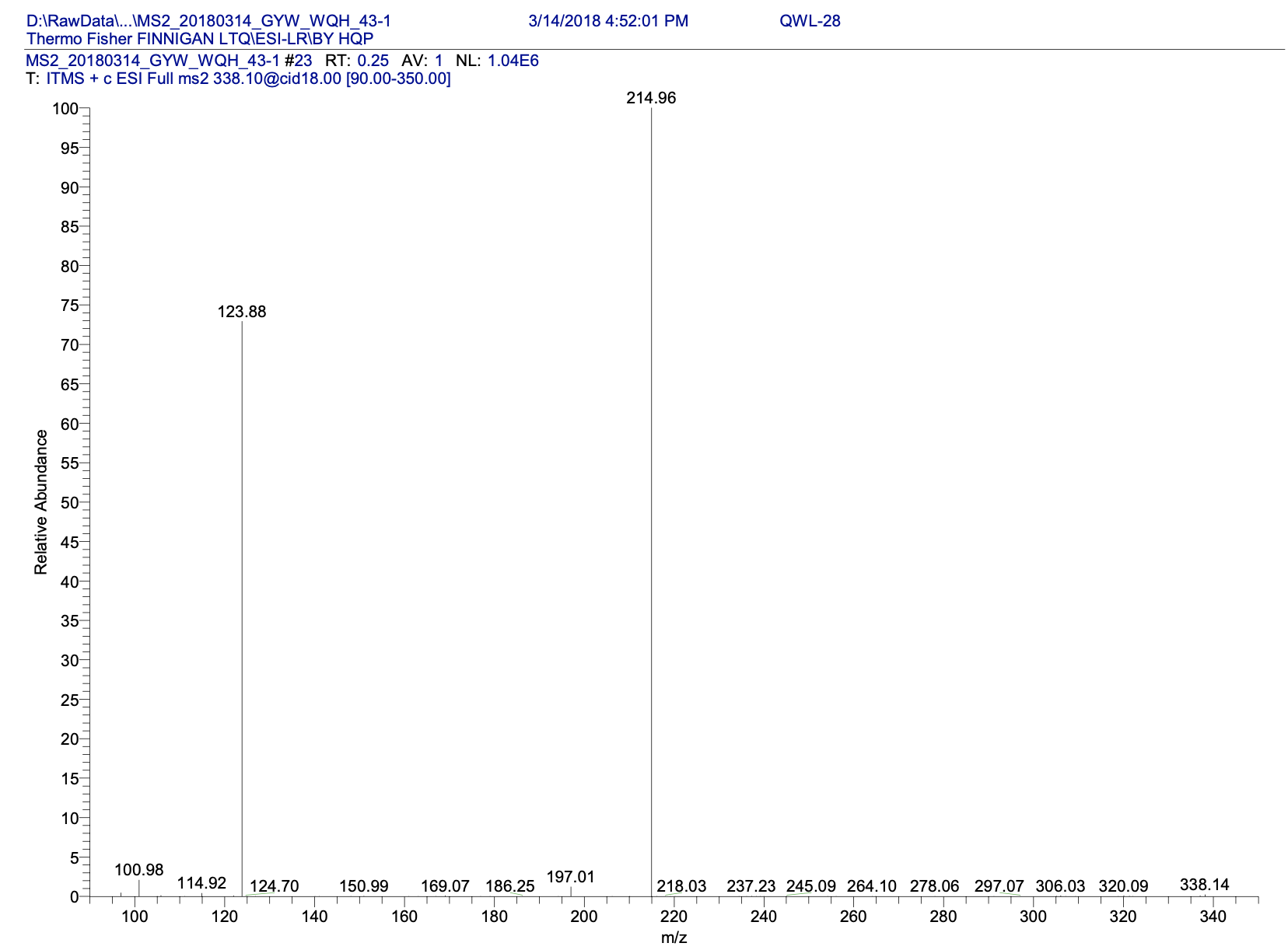


**Supplementary Fig. S10**  ESI-MS^3^ spectrum of **1**.


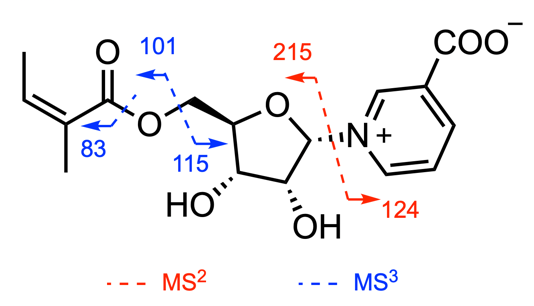

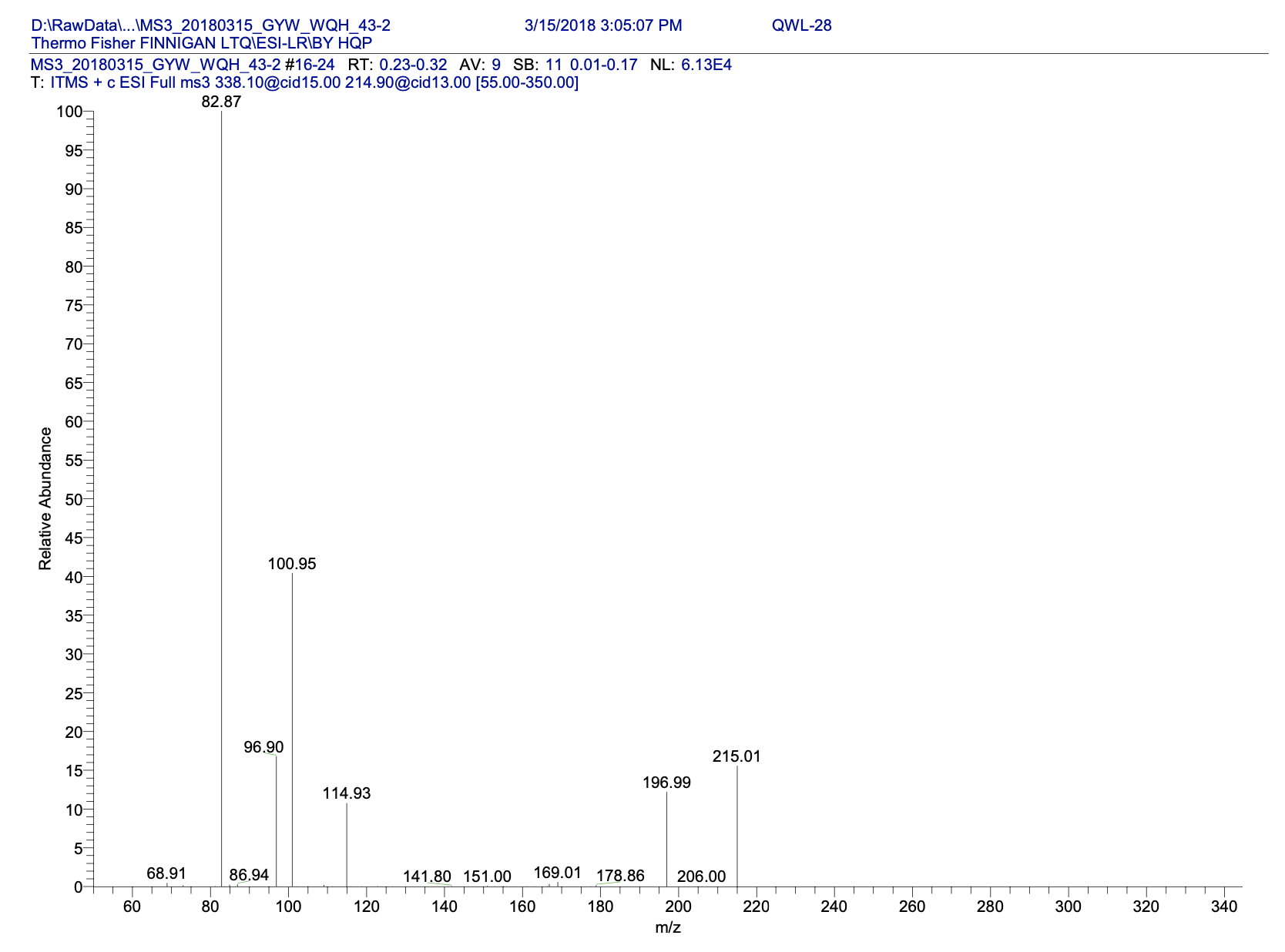


**Supplementary Fig. S11** ESI-MS^3^ spectrum of **1**.


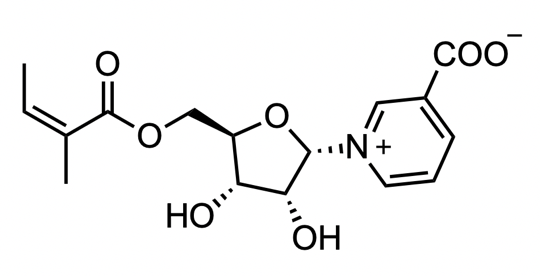

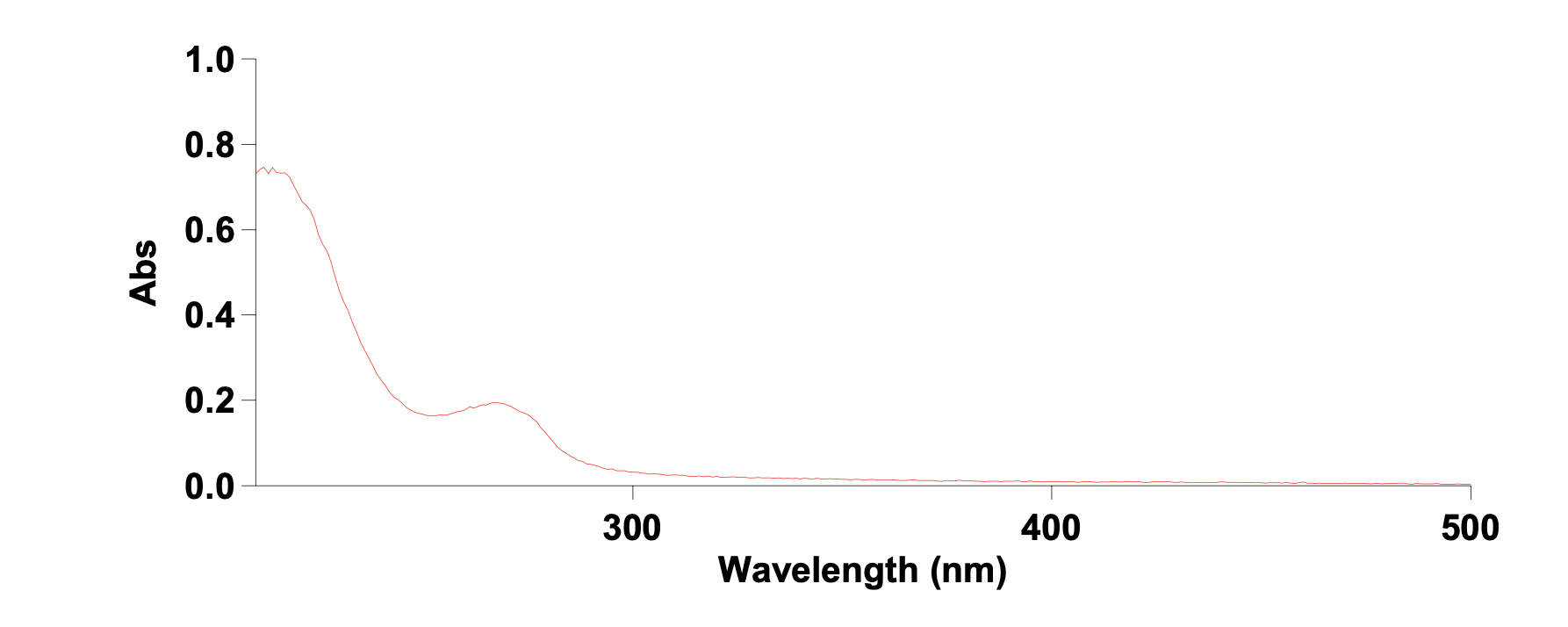

Supplement: Supplementary file 1 — Supplementary file1 (DOCX 3535 KB) [file 42995_2021_96_MOESM1_ESM.docx]
